# The Multidimensional Impact of Gluten-Free Diet Adherence on Quality of Life in Pediatric and Adolescent Celiac Disease: A Systematic Review

**DOI:** 10.3390/children13060722

**Published:** 2026-05-22

**Authors:** Lucía Cascobelo-Águeda, Miguel Garrido-Bueno, María Rodríguez-García, Pastora Tirado-Hernández, Elena Andrade-Gómez, Javier Fagundo-Rivera, Pablo Fernández-León

**Affiliations:** 1 Residencia Valleluz, 04431 Almería, Spain; 2 Centro Universitario de Enfermería Cruz Roja, University of Seville, 41009 Seville, Spain; 3 Research Group PAIDI-CTS-1050: Complex Care, Chronicity and Health Outcomes, Department of Nursing, Faculty of Nursing, Physiotherapy and Podiatry, University of Seville, 41009 Seville, Spain; 4 Huelva District Multiprofessional Teaching Unit for Family and Community Care, National Nurse Specialist Postgraduate Programme, 21003 Huelva, Spain; 5 Department of Surgery and Internal Medicine, Hospital of Calahorra, Rioja Health Service, 26500 La Rioja, Spain; 6 Pre-Departmental Unit of Biomedical Sciences and Health Specialties, University of La Rioja, 26004 Logroño, Spain; 7 Department of Sociology, Social Work and Public Health, Faculty of Labour Sciences, University of Huelva, 21007 Huelva, Spain

**Keywords:** celiac disease, gluten-free diet adherence, pediatric population, adolescents, quality of life, nutritional status, psychological well-being, social impact, family burden, chronic disease management

## Abstract

**Highlights:**

**What are the main findings?**

- Adherence to a gluten-free diet is the key determinant of quality of life in pediatric celiac disease, improving symptoms but also introducing nutritional imbalances and significant social and economic burdens.
- Celiac disease affects multiple domains (physical, psychological, social, and family), being associated with anxiety, social exclusion, dietary challenges, and reduced well-being among both patients and caregivers.

**What are the implications of the main findings?**

- Management should adopt a multidisciplinary approach that goes beyond diet, incorporating psychological support, social integration strategies, and family-centered care.
- Public health policies are needed to reduce economic barriers and improve access to gluten-free foods, alongside education and community interventions to enhance adherence and overall quality of life.

**Abstract:**

Background/Objectives: Celiac disease is a chronic autoimmune disorder triggered by gluten ingestion in genetically predisposed individuals. In children and adolescents, it presents heterogeneously and may negatively affect physical, psychological, and social well-being. Although a strict gluten-free diet is the only effective treatment, it may also impose important dietary, social, and economic burdens. This systematic review aimed to evaluate the impact of celiac disease on the quality of life of affected children and adolescents and their families. Methods: This systematic review was conducted according to PRISMA, AMSTAR 2, and Cochrane Handbook recommendations. Searches were performed in PubMed, Scopus, CINAHL, Web of Science, and PsycINFO for studies published between 2019 and 2026 in English or Spanish. Quantitative, qualitative, and mixed-methods studies on pediatric celiac disease and quality of life were included. Two reviewers independently conducted study selection, data extraction, and risk-of-bias assessment. Due to study heterogeneity, a narrative synthesis was performed. Results: Thirteen studies were included. Children and adolescents with celiac disease generally reported lower quality of life, particularly in emotional, social, and school-related domains. Adherence to a gluten-free diet was an important factor associated with quality of life. Although it improved symptoms, it was also linked to social restrictions, nutritional imbalances, and financial burden. Families also reported stress, lifestyle changes, and reduced well-being. Findings should be interpreted cautiously due to heterogeneity and variability in methodological quality across studies. Conclusions: Current evidence suggests that celiac disease may have a multidimensional impact on the quality of life of pediatric patients and their families. These findings support the need for a comprehensive multidisciplinary approach addressing dietary, psychosocial, and family-related factors.

## 1. Introduction

Celiac disease is a chronic systemic autoimmune disorder triggered by gluten ingestion in genetically predisposed individuals. It is characterized by an immune-mediated inflammatory response affecting the small intestine, leading to villous atrophy and impaired nutrient absorption [1]. Despite this well-established pathophysiology, the clinical presentation of celiac disease remains highly heterogeneous, ranging from classical gastrointestinal symptoms such as diarrhea, abdominal pain, and malabsorption to extraintestinal manifestations including anemia, fatigue, neurological symptoms, and psychological disturbances [2].

The variability in clinical manifestations is particularly evident in pediatric populations. Children may present with growth retardation, delayed development, or nonspecific symptoms such as irritability or lack of concentration, while up to 43% of cases may remain asymptomatic. This heterogeneity complicates early diagnosis and contributes to a significant proportion of undiagnosed cases, estimated to be as high as 70% [3].

The only effective treatment for celiac disease is a lifelong strict gluten-free diet. Although this intervention is effective in resolving symptoms and preventing complications, it represents a substantial burden for patients. Adherence to diet requires constant vigilance, avoidance of cross-contamination, and adaptation to dietary restrictions that may interfere with daily activities [4]. Additionally, gluten-free products are often more expensive and nutritionally imbalanced, typically characterized by higher fat and sugar content and lower levels of fiber and micronutrients [5]. These factors may negatively impact both physical health and overall well-being [2,4].

Quality of life is a multidimensional construct encompassing physical, psychological, and social domains. In children and adolescents, it is influenced not only by health status but also by developmental stage, social interactions, and family environment [6]. Chronic conditions such as celiac disease can significantly alter daily routines, limit social participation, and negatively affect emotional well-being [3].

Previous research has shown that children and adolescents with celiac disease may experience psychological distress, including anxiety and depression, as well as impairments in social functioning [7]. Dietary restrictions, economic burden, and limited access to suitable gluten-free food options have also been associated with reduced quality of life [8]. In addition, family dynamics are increasingly recognized as a key component, as the diagnosis affects not only patients but also caregivers, who may experience increased stress, lifestyle modifications, and financial strain [9].

Despite the growing number of studies in this field, existing evidence remains fragmented and often focused on isolated dimensions of quality of life or single explanatory factors [10,11]. Previous systematic reviews have typically addressed either clinical outcomes, dietary adherence, or specific psychosocial aspects, without fully integrating the combined influence of biological, psychological, social, and family-related determinants in pediatric populations. Particularly, there is still limited synthesis of how gluten-free diet adherence interacts with nutritional patterns, psychosocial functioning, and family burden within a unified quality-of-life framework.

Therefore, the present systematic review addresses this gap by providing an updated and comprehensive synthesis of the multidimensional impact of celiac disease in children and adolescents. Specifically, it integrates evidence on quality of life across physical, psychological, social, and family domains, while also examining key determinants such as dietary adherence, nutritional patterns, and contextual factors. In doing so, this study offers a broader and more integrative perspective, aiming to better inform multidisciplinary clinical management and public health strategies.

## 2. Materials and Methods

### 2.1. Study Design

This study was a systematic review with narrative synthesis that aimed to analyze the impact of celiac disease on the quality of life of children and adolescents. The potential heterogeneity in study designs and outcome measures would make meta-analysis inappropriate, so a narrative synthesis-based approach was adopted while still maintaining the methodological rigor and transparency required for systematic reviews.

This research was conducted in accordance with the methodological standards of the PRISMA statement (Table S1), the AMSTAR 2 tool, and the Cochrane Handbook for Systematic Reviews [12,13,14]. The review was registered prospectively in the Open Science Framework (OSF) as an Open-Ended Registration on 22 April 2026 (registration DOI: https://doi.org/10.17605/OSF.IO/FK4PA). No protocol deviations occurred during the review process, and no amendments were made after the initiation of study screening.

A research question was formulated using the “Person, Exposure, Outcomes’” (PEO) framework, a widely used variant of the “Person, Intervention, Comparison, Outcomes” (PICO) format [15], considering children and adolescents with celiac disease (P), the disease itself (E), and quality of life (O). This formulation guided the development of the search strategy, ensuring direct alignment between the components of the research question and the search terms used.

### 2.2. Search Strategy

The search strategy combined controlled vocabulary and free-text terms, including MeSH descriptors related to “quality of life”, “children”, “adolescents”, and “celiac disease”, in accordance with the research question. Terms were combined using Boolean operators AND and OR, and truncation was applied when appropriate. The general search strategy used was (“quality of life” OR “life quality”) AND (adolescen* OR youth* OR teen* OR child*) AND (“celiac disease” OR “gluten enteropathy” OR celiac).

### 2.3. Information Sources

The information sources included PubMed, Scopus, CINAHL, Web of Science, and PsycINFO.

### 2.4. Elegiblity Criteria

The information search and reference selection process was carried out in February 2026 by two independent reviewers. Interrater agreement was assessed using Cohen’s kappa coefficient, yielding a value of 0.82 [16]. Disagreements were resolved through discussion and, when necessary, by consulting a third reviewer. Filters were applied for English and Spanish language and publication dates between 2019 and 2026.

A preliminary search was performed to identify the available evidence and to define the eligibility criteria. Inclusion criteria comprised original articles with quantitative, qualitative, or mixed-methods designs, published within the defined period, relevant to the study objectives, and available in full text. Exclusion criteria included studies focusing on other autoimmune or gastrointestinal diseases and those not accessible in full text.

### 2.5. Selection Process

**Figure 1:**
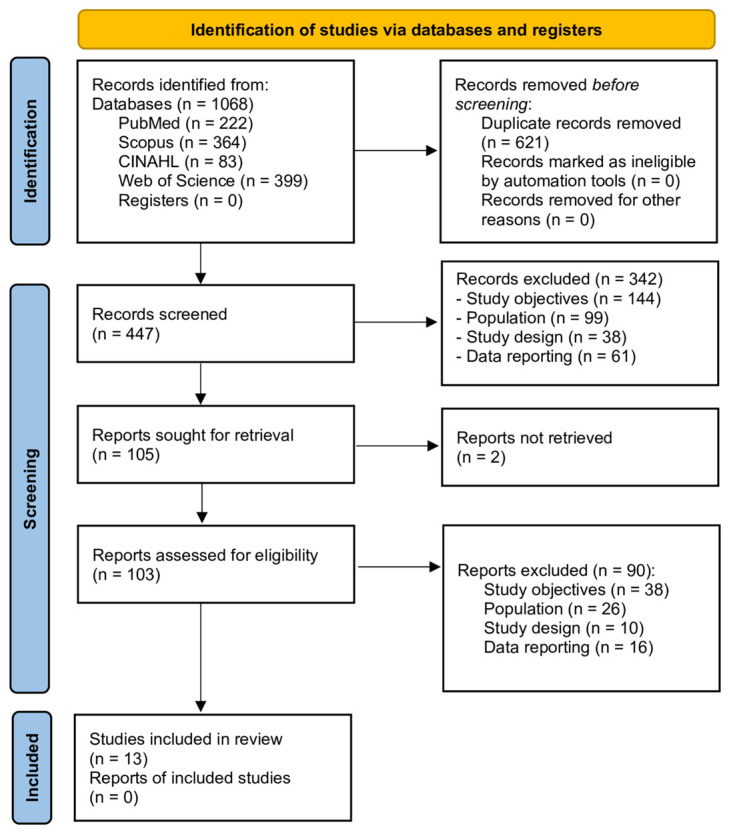
PRISMA flow diagram illustrating the study selection process for the systematic review, including database identification, screening, eligibility assessment, and final inclusion of studies.

Study selection was conducted through a screening process of titles, abstracts, and full texts performed independently by two reviewers using the eligibility criteria. Interrater agreement was assessed using Cohen’s kappa coefficient, yielding values of 0.82 for title and abstract screening and 0.88 for full-text assessment, indicating a high level of agreement [16]. Discrepancies were resolved through discussion and, when necessary, by involving a third reviewer. This process was carried out using the Zotero reference manager version 9.0.3 (Corporation for Digital Scholarship, Falls Church, VA, USA) (Figure 1).

### 2.6. Data Collection Process

Data collection and extraction were carried out in March 2026 by two independent reviewers using a predefined data extraction form. Interrater agreement was assessed using Cohen’s kappa coefficient, yielding a value of 0.85 [16]. Extracted data included study characteristics, sample size and features, measurement instruments, variables analyzed, and main findings. The variables considered included quality of life, adherence to a gluten-free diet, symptomatology, physical and psychological health, and social and family-related factors. Disagreements in data extraction were resolved through consensus or consultation with a third reviewer.

### 2.7. Study Risk-of-Bias Assessment

The risk of bias of the included studies was assessed using the Joanna Briggs Institute (JBI) tools (Joanna Briggs Institute, Adelaide, Australia), selecting the appropriate checklist according to each study design, except for mixed-methods studies, for which the GRAMMS checklist was used due to the lack of a JBI tool for this design [17,18]. This assessment was performed independently by two reviewers. Interrater agreement was evaluated using Cohen’s kappa coefficient, yielding a value of 0.81 [16]. Discrepancies were resolved through consensus or consultation with a third reviewer.

### 2.8. Synthesis Methods

Due to the heterogeneity of the included studies in terms of design, population, measurement instruments, and variables analyzed, a meta-analysis was not performed. Instead, this systematic review employed a structured narrative synthesis, supported by summary tables, to integrate and interpret the findings into four thematic categories:

- Category I: Instruments used to assess quality of life and adherence to a gluten-free diet.
- Category II: Factors that can affect the quality of life of children and adolescents with celiac disease.
- Category III: Physical, psychological and social consequences of celiac disease in affected children and adolescents.
- Category IV: Repercussions of celiac disease on the families of affected children and adolescents.

## 3. Results

### 3.1. Study Selection

A total of 1068 records were initially identified. After removing duplicates (*n* = 621), 447 articles remained and were screened based on title and abstract, leading to the exclusion of 342 studies that did not meet the objectives of the review. A total of 105 articles were assessed for full-text eligibility, of which 2 could not be retrieved. Consequently, 103 studies were evaluated in full text, resulting in a total of 13 studies being included in this review (Figure 1) (Tables S2 and S3).

### 3.2. Risk of Bias in Studies

Two included studies used a mixed-methods approach [19,20] and the other 11 followed a quantitative design. Regarding their quality appraisal, cross-sectional studies scored between 6 and 8, with common limitations in the identification and management of confounding factors. Case–control studies showed high quality (9/10), although strategies to address confounding were often not reported. Cohort studies presented moderate to high quality (7–9/11), with some weaknesses in confounding control and follow-up. Mixed-methods studies showed acceptable quality (5–6/6) (Table S4).

### 3.3. Characteristics of Included Studies

All included studies were published between 2019 and 2024, with the year with the highest publication being 2020 (*n* = 5), followed by 2019 (*n* = 3), 2022 (*n* = 2), 2024 (*n* = 2) and 2023 (*n* = 1). Most studies were conducted in the United States (*n* = 4) and Spain (*n* = 2), followed by India, Serbia, Saudi Arabia, Morocco, Iran, Italy and Jordan (*n* = 1 each). All 13 articles were written in English.

The studies were conducted in pediatric and adolescent populations, with ages ranging mostly from 5 to 18 years. The highest sample was 324 participants [21] and the lowest 16 [20]. All participants shared a diagnosis of confirmed celiac disease.

Quality of life was the main outcome, establishing its relationship with adherence to a gluten-free diet, symptomatology, physical and emotional health, or social and family integration, among others. Finally, regarding data collection techniques, most articles (*n* = 10) used validated questionnaires.

Considerable methodological and clinical heterogeneity was identified across the included studies. Variability was observed in study designs, sample sizes, age ranges, geographical settings, quality-of-life instruments, and definitions of adherence to a gluten-free diet. Outcome assessment methods and psychosocial variables also differed substantially between studies, limiting direct comparability of results and precluding quantitative synthesis through meta-analysis. No subgroup analyses or meta-regression were performed due to the limited number of studies and the heterogeneity of reported outcomes [12,13,14].

### 3.4. Assessing Quality of Life and Adherence to a Gluten-Free Diet

To assess which measurement instruments are used to assess quality of life and adherence to a gluten-free diet in children and adolescents with celiac disease, a total of 10 relevant articles were identified.

Regarding the assessment of quality of life, both generic and specific questionnaires were used. Among the generic instruments, the Pediatric Symptom Checklist (PSC), the Short-Form 36 questionnaire, the KIDSCREEN-52 questionnaire, and the Pediatric Quality of Life (PedsQL) questionnaire were highlighted. Among the specific instruments, the CDDUX questionnaire, the CD-QOL, and the CDPQOOL were identified.

Most studies used instruments such as the KIDSCREEN-52 questionnaire and the PedsQL questionnaire in the generic category, and the CDPQOOL and CD-QOL questionnaires in the specific category.

Concretely, the CDPQOOL questionnaire, used in the studies by Cadenhead et al. [19] and Russo et al. [20], is a validated 17-item survey specifically for pediatric patients with celiac disease. Each question is evaluated on a Likert scale ranging from 0 = never to 4 = almost always. Four domains are assessed: social (*n* = 7), uncertainty (*n* = 3), isolation (*n* = 4), and limitation (*n* = 3). The social domain measures self-esteem, the perception of being a burden, and lack of understanding. Uncertainty items measure concern about the future, college life, and aging with the disease. Isolation items measure whether they feel different from their friends and family, and limitation items measure nervousness about eating at other people’s homes or at social events. The score range goes from 0 to 100, with higher scores indicating better quality of life.

On the other hand, the studies by Al Nofaie et al. [22] and Martín-Masot et al. [23] used the CD-QOL questionnaire, a validated survey specific to patients with celiac disease. It consists of 20 items grouped into 4 domains: limitation (*n* = 9), dysphoria (*n* = 4), health problems (*n* = 5) and inadequate treatment (*n* = 2). The questions were expressed on a five-point Likert scale (1 = not at all, 2 = a little, 3 = moderately, 4 = quite a lot, and 5 = a lot). Since all items were negative except one (“I feel like diet is an insufficient treatment”), the studies reverse-scored these items. Higher total scores indicate better quality of life.

As for the generic questionnaires, the KIDSCREEN-52 was used by the studies of Barrio et al. [24] and Haj-Ahmad et al. [25]. It is a validated cross-cultural generic questionnaire available in 38 languages. It consists of 52 items divided into 10 domains: social acceptance, mood and emotions, physical well-being, psychological well-being, self-perception, school environment, parental relationships and family life, economic resources, autonomy, and social support and peers. Each item is presented on a five-point Likert scale to assess the frequency (never, rarely, sometimes, often, and always) or intensity of an attitude (not at all, slightly, moderately, much, or extremely). Scores can range from 1 to 100. For scores of 1–20 the quality of life is considered “very poor”, 21–40 “bad”, 41–60 “neutral”, 61–80 “good”, and 81–100 “very good”.

Additionally, the Pediatric Quality of Life Questionnaire (PedsQL) was applied in Germone et al. [26] and Stojanovic et al. [27]. There are different versions for three age groups (5–7, 8–12 and 13–18), with each version consisting of 23 items for 4 dimensions (physical (*n* = 5), emotional (*n* = 8), social (*n* = 5) and academic (*n* = 5)). Responses are scored on a five-point Likert scale (0 = never, 1 = almost never, 2 = sometimes, 3 = often, and 4 = always). The scores range from 0 to 100, with higher scores indicating higher quality of life.

On the other hand, in relation to the assessment of adherence to a gluten-free diet, various questionnaires were used, such as Biagi Adherence, the KINDL questionnaire or the CDAT questionnaire, with the latter being used the most in studies.

Cadenhead et al. [19] and Russo et al. [20] included this instrument in their research: a validated seven-item self-administered questionnaire. It consists of two items referring to persistent symptoms (headaches and low energy) and five items on attitudes and behaviors related to gluten exposure. Total scores can range from 7 to 35, with higher scores implying poorer adherence to a gluten-free diet.

Another methodology to analyze adherence to the diet was through a 3-day dietary record, two days during the week and one day on weekends, with the aim of understanding quantities, frequencies, types of foods and their preparation. This approach was applied in the studies conducted by Lionetti et al. [28] and Martín-Masot et al. [23].

### 3.5. Factors Related to Quality of Life of Children and Adolescents with Celiac Disease

Six relevant studies were identified to determine the factors that most affected the quality of life of these patients.

Lionetti et al. [28] evaluated nutritional differences between pediatric patients with celiac disease and healthy children. The findings revealed that daily saturated and total fat intake was much higher in celiac patients, while fiber intake was lower compared to the control group. Both groups exceeded the recommended daily serving of sugary drinks and processed meat. In addition, gluten-free products were shown to provide 34% of sugars, 59% of fiber, and 28% of total fats, contributing to 46% of total daily energy intake. In the same line of research, the study by Martín-Masot et al. [23] reveals that 47.3% of total caloric intake was derived from ultra-processed foods.

The study also shows that children who had been following a gluten-free diet for more than a year experienced more dietary limitations compared to those who had been following it for less than 6 months. Similarly, those who had obtained more than 50% of their daily energy from ultra-processed foods perceived their treatment as less effective, resulting in worse quality of life.

Additionally, Runde et al. [29] showed that all participants consumed processed gluten-free foods. A significant 77% consumed these foods several times a day, while 20% consumed exclusively this type of food. This pattern was observed recurrently in all age groups, although the highest percentage corresponded to children under 12 years old. In addition, the study revealed that 64% of children between 0 and 6 years old and their families wanted to receive dietary advice. However, this desire diminished as time passed since the diagnosis.

On the other hand, Mouslih et al. [21] analyzed how compliance with a gluten-free diet influences the manifestation of clinical symptoms. The authors argued that, before starting the diet, the most frequent symptoms were diarrhea and growth retardation, and type 1 diabetes was the most common comorbidity. In addition, 86.6% of the children were deficient in micronutrients, including vitamin D and iron.

A total of 58.7% of patients followed a gluten-free diet strictly. However, 3.5% never did. Likewise, 87.3% of the participants resolved their clinical symptoms within an average of 6 months after starting the diet, which showed that dietary adherence was related to the remission of symptoms or a decrease in their persistence. It was also correlated with the age of the patients, with adolescents being the least compliant.

Likewise, Chellan et al. [30] evaluated the attenuation of clinical symptoms after 6 months of dietary adherence. The results showed a significant improvement in symptoms such as diarrhea, bloating, anemia and fatigue. Even so, despite the good results, patients reported difficulties in complying with the diet in social settings such as school, weddings and parties. In addition, 40.9% reported feeling different from other children, 13% were not invited to eat due to their diet and 75% had difficulty determining whether the food had gluten or not.

Finally, Cadenhead et al. [19] investigated the impact of diet management on individuals’ quality of life. They divided the sample into two behavioral groups. Those with adaptive behaviors were characterized by greater flexibility, confidence, security, and awareness, and those with maladaptive behaviors were marked by rigidity, avoidance, control, and worry. Thus, they identified that patients with maladaptive behaviors tend to experience more headaches and feel more tired, and to feel lonelier and have problems relating to others. Consequently, it was concluded that patients with maladaptive behaviors had worse quality of life and were older. However, they stressed that it was the diet itself and not age that really affected them, since feeling a constant concern about food was a determining factor that significantly limited their well-being.

### 3.6. Biopsychosocial Consequences of Celiac Disease in Children and Adolescents

Five relevant studies were identified to evaluate the physical, psychological, and social consequences in children and adolescents with celiac disease.

First, Stojanovic et al. [27] observed that, in general terms, children with celiac disease had lower quality of life compared to their healthy peers. This discrepancy manifested itself in multiple domains, covering emotional, social, and school aspects, although without significant differences in physical functioning. However, when analyzing the differences by sex, it was shown that girls, both in the celiac group and in the control group, reported better overall quality of life, except for the emotional aspect. Age was also a relevant factor, as younger children face greater challenges and limitations.

On the other hand, Barrio et al. [24] found that children who maintained high adherence to a gluten-free diet had significantly higher quality of life in all general domains, as demonstrated by the study by Al Nofaie et al. [22]. In contrast, the presence of symptoms derived from non-compliance or economic difficulties that hindered adherence was associated with a decrease in quality of life. Likewise, the authors identified that certain demographic aspects, such as being over 12 years old, being a girl or having had the disease for less than 4 years, were related to a decrease in well-being.

Thus, Yaztappeh et al. [31] observed that children with celiac disease tended to have more psychological problems at school and more difficulty concentrating, in addition to being more likely to experience anxiety, depression, aggression or impulsivity.

Finally, Haj-Ahmad et al. [25] revealed significant differences in quality of life according to sex and the presence of comorbidities.

In general terms of psychological well-being, social support and financial resources, girls had lower scores compared to boys. However, the latter showed significantly lower quality of life in most domains. In addition, the presence of comorbidities had a negative impact particularly on boys, with those with various comorbidities obtaining lower scores in the dimensions of mood and emotions and self-perception. It was also found that girls with growth problems presented more academic and financial challenges. Finally, lack of adherence to the diet was related to challenges in the family environment and more tense relationships with parents.

### 3.7. Impact of Celiac Disease on the Families of Affected Children and Adolescents

Three studies addressing the impact of the diagnosis on families were identified, describing its influence on family functioning and its effect on the quality of life of individuals.

Germone et al. [26] used the Pediatric Quality of Life Questionnaire Family Impact Module (PedsQL FIM) to examine this question. The study argues that caregivers of children with celiac disease have worse quality of life than those without chronically ill children, especially when talking about their children’s health with others and feeling constantly worried about them. However, it also states that family dynamics improved after diagnosis.

In the same vein, Russo et al. [20] observed that family relationships also improved after diagnosis of the disease, adopting new traditions motivated by dietary limitations and strengthening the bond between them. Even so, both parents highlighted the concern of having a child with celiac disease at home, with the tensest moment being right after the diagnosis. In the same research, it was evidenced that mothers experienced more changes in lifestyle and felt the highest dietary burden, while fathers felt guilt for being carriers of the associated gene. On the other hand, siblings developed greater empathy toward others.

On the other hand, Barrio et al. [24] highlighted that parents rated their quality of life as “poor”, especially in the dimensions of autonomy and social and peer support. Likewise, another related factor that had a negative impact on the health of children was economic difficulty, which prevented adequate adherence to a gluten-free diet, the only known treatment for the disease.

## 4. Discussion

This systematic review with narrative synthesis aimed to evaluate the impact of celiac disease on the quality of life of children and adolescents, with particular attention to the factors influencing it, as well as its physical, psychological, social, and family-related consequences. Overall, the findings suggest that celiac disease may have a multidimensional impact on quality of life, with adherence to a gluten-free diet emerging as an important determinant of well-being.

The results of this review are consistent with previous literature, confirming that a gluten-free diet represents both a therapeutic benefit and a significant burden [23,28,29]. While adherence to the diet was associated with symptom resolution in up to 87.3% of patients within approximately six months, it also imposed substantial dietary, social, and economic restrictions [21]. In line with existing evidence, the high cost of gluten-free products and their limited availability were identified as key barriers, contributing to reduced adherence and negatively affecting quality of life [19,30]. This is supported by external reports indicating that individuals with celiac disease may incur substantially higher annual food expenses compared to the general population [32,33].

Nutritional findings further reinforce these concerns. The included studies consistently reported unbalanced dietary patterns among pediatric patients, characterized by high consumption of ultra-processed gluten-free products [23,28,29]. These patterns were associated with increased intake of fats and sugars and lower fiber and micronutrient consumption, which may negatively influence both physical health and perceived treatment effectiveness. These findings are consistent with previous studies highlighting the nutritional inadequacy of many gluten-free products [34].

Beyond dietary imbalance itself, several mechanisms may explain the relationship between gluten-free diet and impaired quality of life. Many commercially available gluten-free products are characterized by high glycemic load, lower fiber content, and reduced micronutrient density, which may contribute to metabolic alterations, persistent fatigue, gastrointestinal dysregulation, and reduced satiety. In addition, micronutrient deficiencies, particularly involving iron and vitamin D, may influence cognitive functioning, emotional regulation, and physical well-being in pediatric populations. These nutritional and metabolic consequences may partially explain why some patients continue to report reduced well-being despite adequate dietary adherence and clinical symptom improvement.

Psychological outcomes emerged as a significant concern among children and adolescents with celiac disease. Included studies reported higher levels of anxiety, depression, concentration difficulties, and social challenges in this population [31], findings that are consistent with previous evidence describing an increased risk of psychological and behavioral disorders [35,36]. Beyond symptom management, the constant need for dietary vigilance and fear of accidental gluten exposure may contribute to anxiety and hypervigilance. In addition, difficulties participating in meals and peer activities were associated with feelings of social exclusion, isolation, and reduced social belonging, while maladaptive coping behaviors and excessive dietary control may further negatively affect emotional well-being and interpersonal functioning [37].

Adherence to the gluten-free diet was consistently identified as a key predictor of quality of life. Higher adherence was associated with improved outcomes across physical, emotional, and social domains, while poor adherence, often linked to social difficulties or economic constraints, resulted in worse outcomes. Social environments such as school, social events, and peer interactions were frequently described as challenging, with a considerable proportion of patients reporting feelings of social exclusion [21,22,24,36]. These findings highlight the importance of considering social integration when managing pediatric celiac disease [38,39].

Another potentially relevant factor is the pathway through which celiac disease is diagnosed. Although this distinction was not consistently addressed in the studies, our review revealed that patients diagnosed after developing symptoms may perceive a more immediate benefit from a gluten-free diet because of symptom improvement. In contrast, patients identified through screening are often asymptomatic at diagnosis and may therefore experience greater challenges in maintaining motivation for long-term dietary adherence. In this sense, previous evidence suggests that screening-detected patients can achieve levels of dietary adherence and quality of life comparable to those diagnosed based on symptoms. However, they may be less likely to attend regular specialist follow-up [40,41,42]. Future studies should systematically examine the influence of diagnostic pathway on long-term adherence, psychosocial adaptation, and quality of life.

Family impact represents another critical dimension. The findings indicate that celiac disease affects not only patients but also their families, altering daily routines, social participation, and emotional well-being. Caregivers were found to experience a greater caregiving burden and lifestyle changes, while economic difficulties further exacerbated stress and reduced overall family quality of life [20,26]. These results are consistent with previous studies describing increased anxiety, social limitations, and financial strain among caregivers of children with chronic conditions [9].

From a family burden perspective, celiac disease should be understood as a chronic condition with evolving effects on family functioning. The post-diagnostic period is often characterized by emotional stress, dietary adaptation challenges, and increased caregiving demands. Although some families develop adaptive coping strategies over time, persistent economic strain, social restrictions, and caregiving responsibilities may continue to negatively affect caregiver well-being and family quality of life [9,43].

Among the studies included in this systematic review, autoimmune comorbidities were addressed only indirectly. Mouslih et al. [21] identified type 1 diabetes as the most common comorbidity in children with celiac disease, while Haj-Ahmad et al. [25] reported that the presence of comorbidities was associated with lower quality-of-life scores, particularly in mood and emotions and self-perception. However, none of the included studies specifically analyzed patients with both celiac disease and type 1 diabetes. Recent evidence suggests that managing these two chronic conditions simultaneously increases dietary complexity, self-management demands, and family burden, which may negatively affect psychosocial well-being and treatment adherence [44,45]. Nevertheless, with appropriate multidisciplinary follow-up, adherence to a gluten-free diet has been associated with satisfactory quality of life and preserved metabolic control [44,46,47]. Future studies should further investigate the impact of autoimmune comorbidities on quality of life in pediatric celiac disease.

### 4.1. Limitations and Strengths

Several limitations should be considered when interpreting the findings of this review. First, substantial methodological and clinical heterogeneity was identified across the included studies, particularly regarding study designs, sample characteristics, quality-of-life instruments, definitions of dietary adherence, and outcome assessment methods [12]. This variability limited the comparability of findings and precluded the performance of a meta-analysis. Consequently, a structured narrative synthesis was conducted to integrate the evidence thematically. Due to the limited number of studies and the heterogeneity of reported outcomes, no subgroup analyses or meta-regression could be performed to further explore potential sources of heterogeneity.

In addition, although most studies demonstrated moderate to high methodological quality according to JBI criteria, most of them adopted quantitative and predominantly cross-sectional designs, limiting causal inference between gluten-free diet adherence and quality of life [48]. Several studies also showed limited adjustment for potentially relevant confounding factors, including socioeconomic status, disease duration, family support, and comorbidities. Variability in methodological quality, reliance on self-reported measures, and potential selection bias may have influenced the robustness and consistency of the reported associations. Furthermore, the restriction to studies published in English or Spanish and the exclusion of articles not available in full text may have introduced selection bias [49]. Finally, the relatively small number of included studies may limit the generalizability and comprehensiveness of the findings.

Despite these limitations, this review presents several strengths. It follows established methodological standards and includes a comprehensive search across multiple databases [12,13,14]. The use of independent reviewers at all stages of the process, together with high interrater agreement, strengthens the reliability of the findings [16]. In addition, the inclusion of studies from different geographical contexts provides a broader perspective on the impact of celiac disease. Furthermore, studies with stronger methodological rigor tended to report more consistent and clinically plausible findings, particularly regarding symptom resolution, emotional well-being, and social functioning.

### 4.2. Implications for Practice, Policy, and Future Research

The findings of this review have important implications for clinical practice and public health. Management of pediatric celiac disease should move beyond a purely biomedical approach and adopt multidisciplinary, family-centered, and community-based strategies that address psychological, social, and nutritional determinants of health [50,51]. In this context, pediatricians, gastroenterologists, dietitians, psychologists, nursing professionals, and school personnel all play an important role in supporting long-term disease management. Routine follow-up should extend beyond monitoring dietary adherence to include nutritional assessment, psychosocial screening, mental health support, and family-focused interventions. Early identification of anxiety, social withdrawal, maladaptive coping behaviors, or caregiver burden may help improve treatment sustainability and prevent long-term deterioration in quality of life, particularly during adolescence and transition into adulthood. These interventions should also be integrated into broader public health initiatives aimed at promoting health literacy, self-management skills, and supportive social environments from the early stages of the disease [52].

From a public health and policy perspective, the economic burden associated with gluten-free products represents a structural barrier to effective disease management. Policies aimed at subsidizing gluten-free foods, improving food labeling regulations, and ensuring equitable access to safe dietary options are essential to reduce health inequalities [53,54]. In addition, school-based interventions and community awareness programs are needed to create supportive environments, reduce stigma, and facilitate social inclusion among children and adolescents with celiac disease [55].

**Figure 2:**
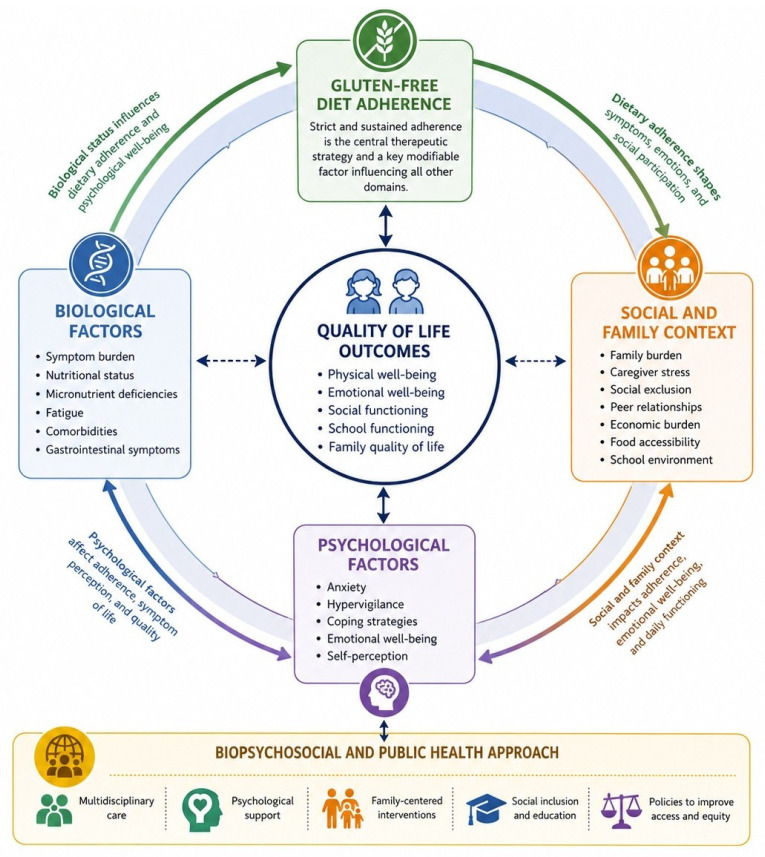
Conceptual biopsychosocial framework of quality-of-life determinants in pediatric celiac disease.

A broader interpretation of these findings supports a conceptual and integrative framework of quality-of-life determinants in pediatric celiac disease, in which biological, psychological, familial, and social factors interact dynamically to influence well-being. This perspective reinforces the need to approach pediatric celiac disease through a biopsychosocial and public health lens rather than focusing exclusively on dietary adherence (Figure 2).

The figure illustrates the dynamic interactions among biological, psychological, and social/family-related factors influencing quality-of-life outcomes in children and adolescents with celiac disease. Adherence to a gluten-free diet is presented as the central modifiable factor affecting symptom control, emotional well-being, social participation, and family functioning. The framework also highlights the importance of multidisciplinary and public health approaches to support comprehensive disease management.

Future research should adopt a population health perspective, focusing on longitudinal and large-scale studies that allow for the identification of social, economic, and environmental determinants influencing quality of life in this population [56]. The development of standardized and culturally sensitive assessment tools is also necessary to improve comparability across studies. Furthermore, greater incorporation of qualitative methodologies would provide deeper insight into the lived experiences of patients and families, supporting the design of more effective, patient-centered, and context-specific public health interventions [48].

## 5. Conclusions

This systematic review suggests that celiac disease may have a substantial and multidimensional impact on the quality of life of children and adolescents, affecting physical, psychological, social, and family domains. However, these findings should be interpreted cautiously due to the heterogeneity of the included studies in terms of design, populations, and assessment instruments, as well as the variability in their internal validity.

Across the included evidence, adherence to a gluten-free diet emerged as an important factor associated with quality of life. Although dietary treatment is essential for symptom control and disease management, several studies reported that it may also impose dietary, social, and economic burdens, potentially contributing to nutritional imbalances and social difficulties that can negatively influence well-being.

Overall, the findings support the importance of a comprehensive and multidisciplinary approach to pediatric celiac disease management that extends beyond dietary treatment and considers the broader psychosocial and family context. Further high-quality and longitudinal research using standardized assessment tools is needed to strengthen the current evidence base.

## Data Availability

No new data were created.

## Supplementary Materials

The following supporting information can be downloaded at: https://www.mdpi.com/article/10.3390/children13060722/s1, Table S1: PRISMA checklist; Table S2: Summary of findings; Table S3: Categorical classification of included studies; Table S4: Critical appraisal of included studies.

## Abbreviations

The following abbreviations are used in this manuscript:

AMSTAR 2	A Measurement Tool to Assess Systematic Reviews 2
CDAT	Celiac Disease Adherence Test
CDPQOOL	Celiac Disease Pediatric Quality of Life Questionnaire
CD-QOL	Celiac Disease Quality of Life Questionnaire
CINAHL	Cumulative Index to Nursing and Allied Health Literature
DOI	Digital Object Identifier
GRAMMS	Good Reporting of A Mixed Methods Study
JBI	Joanna Briggs Institute
KIDSCREEN-52	Cuestionario de Calidad de Vida en Niños y Adolescentes (52 ítems)
MeSH	Medical Subject Headings
PedsQL	Pediatric Quality of Life Inventory
PedsQL FIM	Pediatric Quality of Life Inventory Family Impact Module
PEO	Person, Exposure, Outcomes
PICO	Person, Intervention, Comparison, Outcomes
PRISMA	Preferred Reporting Items for Systematic Reviews and Meta-Analyses
PSC	Pediatric Symptom Checklist
