# Supplementary material for: The Multidimensional Impact of Gluten-Free Diet Adherence on Quality of Life in Pediatric and Adolescent Celiac Disease: A Systematic Review
Source: Children (Basel). 2026 May 22;13(6):722. doi: 10.3390/children13060722 (PMC13297346; doi:10.3390/children13060722)
Supplement: Supplementary file 1 [file children-13-00722-s001.zip › Supplementary 2 Summary of Findings.pdf]

Supplementary Table S2
Summary of Findings

Author, year and country	Methodology/Sample	Objectives	Main results
Al Nofaie et al. (2019) Saudi Arabia [22]	Case-control study of pediatric celiac patients (between 9-18 years old), Saudi.<br><br>The sample consisted of 111 patients with celiac disease and 243 controls (41 were previously excluded thanks to the CD screening questionnaire)<br><br>The variables that were measured were adherence to the diet and quality of life.<br><br>Adherence to the gluten-free diet was measured by combining the KINDL adherence questionnaire and demonstrating the decrease in tissue transglutaminase antibody levels to near-normal levels.<br>Quality of life was measured with the generic Short-Form questionnaire (SF-36) for all participants and the specific quality of life questionnaire (CD-QOL) for patients with celiac disease.	"Reporting on the quality of life of Saudi children and adolescents with celiac disease"	Women made up 56.8% of the total participants.<br><br>The generic questionnaire showed no significant differences between patients and controls in quality of life, except in the general health domain. However, men scored lower in the domains of changes in health, physical functioning and emotional well-being.<br><br>The specific questionnaire reported that 79.3% of patients showed a good or excellent score in the domains evaluated.<br><br>Regarding quality of life as a function of diet adherence, it was shown that children with better adherence had a significantly better quality of life in all general domains.
Barrio et al. (2020) Spain [24]	Two questionnaires were used to measure the quality of life of children with celiac disease from Madrid, between 8 and 18 years old.<br><br>The generic KIDSCREEN-52 questionnaire, completed by 255 children and 387 parents.	"To compare the perception of health-related quality of life and related factors in Spanish celiac children and their parents, using a generic and a specific questionnaire and to	Both rated their quality of life as "poor" in the dimensions of autonomy and social support and equal, although parents scored, in general, higher than boys.<br><br>Based on related factors, having non-treatment symptoms and having economic or social difficulties adhering to the diet were shown to decrease quality of life scores. While children with adherence to the diet scored having a higher

	The specific questionnaire of the EC DUX (CDDUX), answered by 266 children and 428 parents.<br>In addition, linear regression models were fitted to measure the association of demographic and clinical factors with scores.	evaluate the correlation between them"	quality of life.<br>In addition, age over 12 years of age (dimensions of moods, psychological, physical and social well-being) was related;being a girl (psychological and physical well-being dimensions) and time since diagnosis less than 4 years (psychological well-being and self-esteem dimensions) with a decrease in quality of life.<br><br>Finally, it was observed that a better quality of life, especially in the diet, is associated with a better perception of health.<br><br>The correlation between the two questionnaires was poor, being worse in children than in parents.
Cadenhead et al. (2019) USA [19]	Mixed-methods cross-sectional study of 30 adolescents (aged 13-17 years) diagnosed with celiac disease with self-reported duodenal biopsy at least one year earlier at Columbia University Medical Center.<br><br>Four variables were assessed: demographic aspects and medical history of the participants, quality of life, adherence to the gluten-free diet and eating patterns.<br><br>Adherence to the diet was measured with the celiac dietary adherence test (CDAT), in addition to collecting 3 dietary records of 24 hours over 1 month, to determine the amount and frequency of exposure to gluten and thus, the level of attention.<br>Quality of life was measured with the	"Understanding adolescent approaches to managing a gluten-free diet and how it is associated with quality of life"	80% of the participants were women, with a mean age of 15.6 years.<br><br>The sample was divided into adolescents with adaptive or maladaptive thoughts and behaviors. Adaptive behaviors were characterized by greater flexibility (vs. rigidity), confidence (vs. avoidance), security (vs. control), and conscientiousness (vs. worry).<br>It was identified that participants with maladaptive behaviors (53.3%) had a worse quality of life and tended to be older. However, after considering age, they stressed that it is the diet itself, and not age that really affects them, especially worrying about food and feeling limited.<br><br>Thus, although everyone followed the gluten-free diet, adolescents with disadaptive behaviors tended to have more headaches, feel more tired and extremely vigilant, although the latter was not statistically relevant. They also have problems relating to others and feel lonely. Although, they are less likely to eat gluten-containing foods on purpose.

	Celiac Disease-Specific Pediatric Quality of Life Questionnaire (CDPQOL)<br>The approach to diet management was made through a semi-structured interview guided by a psychosocial rubric.		
Chellan et al. (2019) India [30]	Prospective follow-up study of 44 pediatric celiac patients (between 2 and 12 years) in northern India.<br><br>The variables studied were quality of life and the impact of the gluten-free diet.<br>Quality of life was measured with a generic questionnaire, pediatric symptom checklist (PSC), and a disease-specific questionnaire (including questions about dietary adherence, parental behavior and perception, food safety, and child feelings).<br><br>The pediatric symptom checklist was administered at the start of the gluten-free diet and 6 months after starting it; while the specific questionnaire only after 6 months have passed.	"To study the quality of life in pediatric patients with celiac disease and the effect of a gluten-free diet"	The mean age of the group was 6.03 years and 68.2% were boys.<br><br>After 6 months of adherence to the gluten-free diet, an improvement in the clinical symptoms of the disease was shown, such as diarrhea, anemia, abdominal distension or fatigue. However, there were barriers to dietary adherence especially at school, weddings and parties.<br><br>In addition, 40.9% felt different from other children, 13.6% felt that they were not invited to eat due to their diet and 75% had difficulty determining whether the food had gluten or not.
Germone et al. (2022) USA [26]	The sample had a total of 225 pediatric patients (between 5 and 18 years old) with CD and their caregivers (n= 246)<br><br>The variables to be studied were quality of life and family impact.<br><br>Quality of life was measured by paediatric quality of life (PedsQL),	"To evaluate the impact of celiac disease and gluten-free diet on quality of life in celiac children and their caregivers"	Children with CD reported significantly lower quality of life than controls in all domains, and in the domains of general social and psychosocial functioning compared to children with other organic gastrointestinal conditions. However, the caregiver's report did not allege any decrease, stating that he was unaware of his children's concerns.

	completed by children and caregivers Family impact was measured by the Family Impact Module (FIM), completed only by caregivers.<br><br>The results were compared with previously published results from a cohort with organic gastrointestinal disease (n= 298), a healthy cohort (n= 936), and their caregivers.		Caregivers' outcomes showed poorer quality of life than controls in the communication and worry domains, but better in social functioning and family relationships.
Haj-Ahmad et al. (2024)<br>Jordan [25]	Cross-sectional study of 126 children (between 8-18 years old) with celiac disease.<br><br>Quality of life was assessed through the Kidscreen-52 questionnaire.	"To assess the quality of life among Jordanian children with celiac disease"	55.6% were women and the mean age of the sample was 13.4 years. Approximately 61.1% followed the gluten-free diet strictly, 67.5% had comorbid diseases, and 34.1% had growth problems.<br><br>Women scored worse than men on psychological well-being, social support, and financial resources. However, boys had significantly lower quality of life in a higher number of domains.<br><br>Since 67.5% of the patients had comorbid diseases, the quality of life of these patients was compared with those who do not. Thus demonstrating that men with more than one chronic disease presented lower scores in the dimensions of mood and emotions and self-perception.<br><br>The quality of life of those who had growth problems was also compared, showing that women faced more academic and financial challenges.<br><br>Thus, those who did not follow a strict gluten-free diet also reported more strained relationships with their parents and challenges in the family environment.
Lionetti et al. (2020)<br>Italy [28]	Prospective case-control study of celiac children (between 4 and 16 years old)	"Assess nutritional status, dietary intake, and adherence to	No significant differences were found between patients and controls in anthropometric measurements and energy expenditure, however, there were significant differences in

	who have been following a gluten-free diet for more than 2 years.<br><br>The sample consisted of 120 children with CD (72 women) and 100 healthy children (56 women).<br>The variables studied were anthropometric measurements, physical activity and food intake.<br><br>Anthropometric measurements were evaluated using BMI.<br>Physical activity/energy expenditure through a questionnaire.<br>Feeding was measured through a 3-day diary, 2 during the week and 1 on the weekend. Including all foods from all meals with a detailed description of the quantity, recipes, and brand of packaged foods consumed.<br>Adherence to the Mediterranean diet was also evaluated, with the KIDMED index.	dietary recommendations"	dietary intake.<br>Daily intake of saturated and total fat was much higher in CD patients, and fiber intake was lower compared to the control group. The daily intake of carbohydrates is also lower, as well as that of simple sugars.<br><br>However, none of the groups reached the number of servings recommended by the Italian food pyramid; recommended by the Italian Society of Pediatrics, for legumes, vegetables, eggs and fish, while they surpassed the consumption of sugary drinks and meat.<br><br>In addition, it was shown that products specifically formulated gluten-free provide 59% of the fiber, 34% of the sugars, 73% of the carbohydrates and 28% of the total fats, providing 46% of the total daily energy.<br><br>The KIDMED index showed suboptimal adherence in both groups.
Martín-Masot et al. (2022)<br>Spain [23]	The sample consisted of 58 celiac children (between 7 and 18 years old).<br><br>They were divided into 2 groups:<br>Gluten-free diet less than 6 months (n= 18)<br>Gluten-free diet older than 12 months (n= 37)<br><br>The variables studied were sociodemographic and clinical characteristics, dietary intake and quality of life.	"To evaluate the influences of the gluten-free diet and the consumption of ultra-processed foods on parents' perception of the quality of life of children with celiac disease"	Children who had followed a gluten-free diet for more than 12 months had greater limitations than those who followed it for 6 months.<br><br>On average , 47.3% of total energy intake came from ultra-processed foods.<br><br>In addition, it was found that children who obtained more than 50% of their daily energy from ultra-processed foods perceived more limitations and obtained less effective treatment, tending to have a lower quality of life.

	Sociodemographic characteristics were collected through a self-administered survey<br>Quality of life was measured with the Celiac Disease Quality of Life Survey (CD-QOL)<br>Dietary intake, through a 3-day record, 2 during the week and 1 on the weekend. Including all foods from all meals, as well as the portions, recipes and brands of packaged foods.		
Mouslih et al. (2023) Morocco [21]	Retrospective cohort study of 324 children with CD.<br><br>The study variables were: sociodemographic data, effect of gluten before diagnosis and clinical symptoms at the time of diagnosis, as well as the degree of adherence to the diet after diagnosis.<br><br>For adherence to the diet, a routine retrospective follow-up was performed at 6 months, 12 and 18 months. It was evaluated using a gluten consumption scale.	"To assess the impact of gluten-free diet adherence on clinical symptoms in children with celiac disease"	60.8% of the sample were women.<br><br>Before starting the diet, the most frequent symptoms were diarrhea and delayed growth. In addition, they found that the most common associated disease was type 1 diabetes, and 86.6% of children had micronutrient deficiencies, as well as vitamin D and iron deficiency.<br><br>58.7% of the children strictly followed the diet, while 3.5% never did. This was directly related to the age of the child, with adolescents being the least compliant, and to the type of clinical symptoms present.<br><br>Adherence to the diet was also related to the disappearance of symptoms, persistence and the appearance of complications. 87.3% of the children resolved their clinical symptoms in an average of 6 months.
Runde et al. (2020) USA [29]	The sample consisted of 100 children with CD (aged 18 years or younger).<br>They were divided into three groups: Children from 13 to 18 years old; Children from 7 to 12 years old; Children from 0 to 6 years old	"Assessing the Dietary Preferences of a Cohort of Celiac Children"	63 % were girls and the mean age of the study was 11.85 years.<br><br>It was shown that all participants consumed processed gluten-free foods, 77% several times a day and 20% only ate these types of foods.

	All completed a cross-sectional survey, anonymously, on diet compliance.		This pattern was repeated in all age groups, however, the percentage was higher in children under 12 years of age compared to older children.<br><br>76% of participants identified convenience as their main motivation.<br><br>This study also showed that almost 64% of children between 0 and 6 years of age and their families were interested in dietary advice, while in patients between 13 and 18 years of age the interest was reduced to less than 5%, revealing that the desire to receive such advice decreased over time from diagnosis.
Russo et al. (2020) USA [20]	A mixed convergent and parallel methods study, mainly with a qualitative approach, of 16 families with at least one child (between 8 and 18 years old) with celiac disease diagnosed by duodenal biopsy at least 1 year earlier, following the gluten-free diet, in New York City.<br><br>Finally, the sample consisted of 16 children, 31 parents and 24 siblings.<br><br>The following variables were evaluated:<br>The overall impact of the disease on the family (through semi-structured interviews)<br>Demographic variables and medical history<br>Adherence to the gluten-free diet<br>Quality of life of children<br>Caregiver concern<br><br>Diet adherence was measured with the	"Examining the effects of celiac disease on the whole family"	In general, the families were two-parent, with a high socioeconomic level, all the mothers had university studies and 100% of the fathers worked outside the home.<br>The mean age of the children was 12.6 years and 56.3% were girls, and had had an average of 5.1 years with the disease.<br>In general, adherence to the gluten-free diet was rated as high in both questionnaires.<br><br>Quality of life was also rated as high, although girls more than boys; and children with more severe symptoms when exposed to gluten scored lower than those with mild symptoms or symptoms.<br><br>Caregiver concern questionnaire scores indicated significant changes between before and after diagnosis for both parents, with the time of greatest concern being right after diagnosis.<br><br>Mothers expressed more lifestyle changes and felt the heaviest dietary burden, while fathers felt guilty for being

	Celiac Disease Adherence Test (CDAT), completed by parents on behalf of their children, and with the Biagi Adherence Questionnaire, completed as the previous one and also by the children themselves. Both scales were reformulated so that parents could respond.<br><br>Quality of life was measured using the Paediatric Quality of Life for Celiac Disease (CDPQOL) questionnaire<br><br>The Ferretti caregiver questionnaire was used for caregiver concern and the impact of celiac disease. The questionnaire was slightly modified to avoid questions that were not applicable to children.		carriers of the associated gene, siblings reported that they had developed more empathy for others, and both fathers and siblings felt the dietary limitations in restaurants and at home. However, they appreciated positive aspects such as having to be more creative in the kitchen or having to create new family traditions.
Stojanovíc et al. (2019) Serbia [27]	Observational and cross-sectional cohort study.<br><br>The sample included 116 children (aged 5 to 18 years) with CD who had been on a gluten-free diet for at least one year, and 116 healthy controls.<br><br>The main study variable was quality of life, measured thanks to the Pediatric Quality of Life Questionnaire (PedsQL). The questionnaire was modified for the 3 age groups (5-7 years, 8-12 and 13-18 years)	"To examine the quality of life of children and adolescents with celiac disease and to obtain a representation of physical, mental and social functioning impairments compared to healthy children"	In general, the group of celiac children showed a worse quality of life compared to the control group.<br><br>In relation to sex, girls in both groups reported having a better quality of life, except in the domain of emotional functioning; and with respect to age, the youngest presented more difficulties and limitations.<br><br>The group with celiac disease reported a lower score in almost all domains (emotional, social, and school) except for the physical functioning domain, where there were no significant differences.
Yaztappeh et al. (2023) Iran [31]	Cross-sectional case-control study of 105 children with CD (between 6 and 12) and 105 healthy children, and their parents.	"To evaluate and compare the quality of life in children with CD and healthy children"	The mean age of both groups was 9.7 years.<br><br>The study found that parents tended to have a more positive view of their children's quality of life than they did

Quality of life was measured with the Kid-KINDL quality of life questionnaire, completed by children and parents; and the Pediatric Symptom Checklist (PSC), completed by parents only.

Note. Authors' own elaboration

themselves. On the other hand, children felt more confident about themselves than parents perceived.

It was also observed that children with CD tended to have more psychological problems, at school and more difficulty concentrating. In addition, they scored higher on internalizing and externalizing symptoms, indicating that they are more likely to experience anxiety, depression, aggression, or impulsivity.
Despite this, there were no significant differences between the two groups in terms of overall quality of life or physical well-being, family and friends.
