# Supplementary material for: The Multidimensional Impact of Gluten-Free Diet Adherence on Quality of Life in Pediatric and Adolescent Celiac Disease: A Systematic Review
Source: Children (Basel). 2026 May 22;13(6):722. doi: 10.3390/children13060722 (PMC13297346; doi:10.3390/children13060722)
Supplement: Supplementary file 1 [file children-13-00722-s001.zip › Supplementary 3 Categorical Classification.pdf]

Supplementary Table S3

Categorical Classification of Included Studies

Study	Category I	Category II	Category III	Category IV
Al Nofaie et al. [22]	YES			YES
Barrio et al. [24]	YES	YES		YES
Cadenhead et al. [19]	YES		YES	
Chellan et al. [30]	YES		YES	
Germone et al. [26]	YES	YES		
Haj-Ahmad et al. [25]	YES			YES
Lionetti et al. [28]			YES	
Martín-Massot et al. [23]	YES		YES	
Mouslih et al. [21]			YES	
Runde et al. [29]			YES	
Russo et al. [20]	YES	YES		
Stojanovic et al. [27]	YES			YES
Yaztappeh et al. [31]	YES			YES

Note. Authors' own elaboration.
