# Supplementary material for: The Multidimensional Impact of Gluten-Free Diet Adherence on Quality of Life in Pediatric and Adolescent Celiac Disease: A Systematic Review
Source: Children (Basel). 2026 May 22;13(6):722. doi: 10.3390/children13060722 (PMC13297346; doi:10.3390/children13060722)
Supplement: Supplementary file 1 [file children-13-00722-s001.zip › Supplementary 4 JBI.pdf]

Supplementary Table S4

Critical Appraisal for Included Studies

Cross Sectional Studies

Study	1	2	3	4	5	6	7	8	Total
Barrio et al. [24]	YES	YES	YES	YES	YES	YES	YES	YES	8
Germone et al. [26]	YES	YES	YES	YES	NO	NO	YES	YES	6
Haj-Ahmad et al. [25]	YES	YES	YES	YES	YES	NO	YES	YES	7
Martín-Masot et al. [23]	YES	YES	YES	YES	YES	YES	YES	YES	8
Runde et al. [29]	YES	YES	YES	YES	NO	NO	YES	YES	6
Stojanovic et al. [27]	YES	YES	YES	YES	NO	NO	YES	YES	6

Note. Authors' own elaboration. 1: Were the criteria for inclusion in the sample clearly defined?; 2: Were the study subjects and the setting described in detail?; 3: Was the exposure measured in a valid and reliable way?; 4: Were objective, standard criteria used for measurement of the condition?; 5: Were confounding factors identified?; 6: Were strategies to deal with confounding factors stated? ; 7. Were the outcomes measured in a valid and reliable way?; 8. Was appropriate statistical analysis used?

Case Control Studies

Study and total	1	2	3	4	5	6	7	8	9	10	Total
Al Nofaie et al. [22]	YES	YES	YES	YES	YES	YES	NO	YES	YES	YES	9
Lionetti et al. [28]	YES	YES	YES	YES	YES	YES	NO	YES	YES	YES	9
Yaztappeh et al. [31]	YES	YES	YES	YES	YES	YES	NO	YES	YES	YES	9

Note. Authors' own elaboration. 1: Were the groups comparable other than the presence of disease in cases or the absence of disease in controls?; 2: Were cases and controls matched appropriately?; 3: Were the same criteria used for identification of cases and controls?; 4: Was exposure measured in a standard, valid and reliable way?; 5: Was exposure measured in the same way for cases and controls?; 6: Were confounding factors identified?; 7: Were strategies to deal with confounding factors stated?; 8: Were outcomes assessed in a standard, valid and reliable way for cases and controls?; 9: Was the exposure period of interest long enough to be meaningful?; 10: Was appropriate statistical analysis used?

Cohort Studies

Study	1	2	3	4	5	6	7	8	9	10	11	Total
Chellan et al. [30]	YES	YES	YES	YES	NO	YES	YES	YES	YES	NO	YES	9
Mouslih et al. [21]	YES	YES	YES	NO	NO	YES	YES	YES	NO	NO	YES	7

Note. Authors' own elaboration. 1: Were the two groups similar and recruited from the same population?; 2: Were the exposures measured similarly to assign people to both exposed and unexposed groups?; 3: Was the exposure measured in a valid and reliable way?; 4: Were confounding factors identified?; 5: Were strategies to deal with confounding factors stated?; 6: Were the groups/participants free of the outcome at the start of the study (or at the moment of exposure)?; 7: Were the outcomes measured in a valid and reliable way?; 8: Was the follow up time reported and sufficient to be long enough for outcomes to occur?; 9: Was follow up complete, and if not, were the reasons to loss to follow up described and explored?; 10: Were strategies to address incomplete follow up utilized?; 11: Was appropriate statistical analysis used?

Mixed-Methods Studies

Study	1	2	3	4	5	6	Total
Cadenhead et al. [19]	YES	YES	YES	YES	NO	YES	5
Russo et al. [20]	YES	YES	YES	YES	YES	YES	6

Note. 1: Describe the justification for using a mixed methods approach to the research question; 2: Describe the design in terms of the purpose, priority and sequence of methods; 3: Describe each method in terms of sampling, data collection and analysis; 4: Describe where integration has occurred, how it has occurred and who has participated in it; 5: Describe any limitation of one method associated with the presence of the other method; 6: Describe any insights gained from mixing and integrating methods
